# Molecular analysis of lymphoid tissue from rhesus macaque rhadinovirus-infected monkeys identifies alterations in host genes associated with oncogenesis

**DOI:** 10.1371/journal.pone.0228484

**Published:** 2020-02-04

**Authors:** Ryan Douglas Estep, Aparna N. Govindan, Minsha Manoharan, He Li, Suzanne S. Fei, Byung S. Park, Michael K. Axthelm, Scott W. Wong

**Affiliations:** 1 Vaccine and Gene Therapy Institute, Oregon Health & Science University, Beaverton, Oregon, United States of America; 2 Bioinformatics & Biostatistics Core, Oregon National Primate Research Center, Oregon Health & Science University, Beaverton, Oregon, United States of America; 3 Oregon Health & Science University-Portland State University School of Public Health, Portland, Oregon, United States of America; 4 Knight Cancer Institute, Oregon Health & Science University, Portland, Oregon, United States of America; 5 Division of Pathobiology and Immunology, Oregon National Primate Research Center, Beaverton, Oregon, United States of America; 6 Department of Molecular Microbiology and Immunology, Oregon Health & Science University, Portland, Oregon, United States of America; UPMC Hillman Cancer Center, UNITED STATES

## Abstract

Rhesus macaque (RM) rhadinovirus (RRV) is a simian gamma-2 herpesvirus closely related to human Kaposi’s sarcoma-associated herpesvirus (KSHV). RRV is associated with the development of diseases in simian immunodeficiency virus (SIV) co-infected RM that resemble KSHV-associated pathologies observed in HIV-infected humans, including B cell lymphoproliferative disorders (LPD) and lymphoma. Importantly, how *de novo* KSHV infection affects the expression of host genes in humans, and how these alterations in gene expression affect viral replication, latency, and disease is unknown. The utility of the RRV/RM infection model provides a novel approach to address these questions *in vivo*, and utilizing the RRV bacterial artificial chromosome (BAC) system, the effects of specific viral genes on host gene expression patterns can also be explored. To gain insight into the effects of RRV infection on global host gene expression patterns *in vivo*, and to simultaneously assess the contributions of the immune inhibitory viral CD200 (vCD200) molecule to host gene regulation, RNA-seq was performed on pre- and post-infection lymph node (LN) biopsy samples from RM infected with either BAC-derived WT (*n* = 4) or vCD200 mutant RRV (*n* = 4). A variety of genes were identified as being altered in LN tissue samples due to RRV infection, including cancer-associated genes activation-induced cytidine deaminase (*AICDA*), glypican-1 (*GPC1*), CX3C chemokine receptor 1 (*CX3CR1*), and Ras dexamethasone-induced 1 (*RasD1*). Further analyses also indicate that GPC1 may be associated with lymphomagenesis. Finally, comparison of infection groups identified the differential expression of host gene thioredoxin interacting protein (*TXNIP*), suggesting a possible mechanism by which vCD200 negatively affects RRV viral loads *in vivo*.

## Introduction

Rhesus macaque rhadinovirus (RRV) is a rhesus macaque (RM) gamma-2 herpesvirus that is closely related to human herpesvirus 8 (HHV-8)/Kaposi’s sarcoma-associated herpesvirus (KSHV), and causes diseases in simian immunodeficiency virus (SIV)-infected RM similar to those observed in humans co-infected with KSHV and HIV, including B cell lymphoproliferative disorders (LPD), non-Hodgkin’s lymphoma (NHL), multicentric Castleman disease (MCD), and a Kaposi’s sarcoma (KS)-like mesenchymal proliferative lesion termed retroperitoneal fibromatosis (RF) [1, 2]. Further, similar to KSHV, RRV naturally infects B cells *in vivo*, where the virus is capable of establishing a latent infection [3]. In regards to KSHV, little is known about the impact of *de novo* infection on host gene expression patterns in humans, or how such alterations in specific tissues may ultimately affect viral pathogenesis and disease development. Thus, use of an *in vivo* infection model that utilizes a phylogenetically related primate virus and its natural host is critical to address these questions, and to help shed light onto mechanisms that may affect infection, replication, immune regulation, and disease development in KSHV-infected humans. Fortunately, RRV infection of naïve RM provides a well-established primate model system with which to assess the effects of gamma-2 herpesvirus infection on alterations in host gene expression in a variety of tissues relevant to both RRV and KSHV disease development. In general, analysis of the effects of RRV infection on host gene expression patterns in specific tissue samples can provide critical information as to how infection regulates genes or gene pathways within an infected host that may be important for the virus to successfully establish an infection and promote disease development, and alternatively, can also provide information as to how the infected host regulates and controls infection.

Due to the utility of the RRV BAC system, it is also possible to generate recombinant viruses that can be used to assess the effects of individual viral genes and viral factors on infection and host gene expression profiles *in vivo*. RRV possesses a variety of genes that are positional and functional homologues of KSHV genes believed to be directly involved in viral pathogenesis, all of which are capable of altering the cellular and host environment during infection [4–8]. One KSHV/RRV-encoded factor of particular interest is the viral CD200 (vCD200) protein. Several DNA viruses, including members of the herpesvirus and poxvirus families, have been found to encode vCD200 molecules, which themselves are homologues of cellular CD200 [9–12]. CD200 is an immunomodulatory molecule that binds to cellular CD200 receptor (CD200R), inducing inhibitory signals in immune cells expressing the receptor [13, 14]. KSHV vCD200 is the most extensively studied vCD200 molecule, and has been found to induce signaling that inhibits the activation of a variety of immune cells expressing CD200R, including macrophages, basophils, neutrophils, and T cells [11, 15–17]. Despite this, *in vivo* studies of KSHV vCD200 functionality have not been performed. In previous *in vitro* studies, we demonstrated that RRV vCD200 is functionally similar to KSHV vCD200, and is capable of inhibiting the activation of CD200R+ macrophages [6]. Further, through the utilization of our RRV/RM infection model, and a mutant RRV BAC-derived virus lacking expression of vCD200 (vCD200 N.S.), we have also analyzed the function of RRV vCD200 in infected RM [18], providing the first assessment of the effects of a gamma-2 herpesvirus vCD200 molecule on immune regulation, viral replication, and pathogenesis *in vivo*. Through these studies, RRV vCD200 was determined to affect immune responses at early times post-infection (pi), specifically inhibiting CD4 and CD8 T cell activation at days 7 and 21 pi. Further, despite the development of higher RRV-specific T cell responses in these animals, vCD200 N.S.-infected RM also displayed higher average peak viral loads in both whole blood (WB) and LN biopsy samples, when compared to RM infected with WT BAC RRV. Overall, the observed differences in viral loads between vCD200 N.S. and WT BAC infection groups were not anticipated, given that earlier RRV-specific T cell responses developed in vCD200 N.S.-infected RM in the absence of the expression of the immunosuppressive vCD200 molecule. These observations led to our interrogation of the role of RRV vCD200 in the regulation of cellular genes in infected RM tissue that might affect RRV replication, either in addition to, or aside from, the effects of this molecule on immune regulation. Further, as LN represent a natural site of RRV and KSHV tropism, and the site where most B cell lymphomas develop [1, 2, 19], examination of the overall effects of RRV-infection on cellular gene expression profiles in infected LN tissue provides a molecular approach to obtain novel information regarding the role of both RRV and KSHV in immune regulation and disease development *in vivo* at defined time point(s) pi.

To determine the effects of RRV infection and RRV vCD200 expression on cellular gene expression patterns *in vivo*, we initiated RNA-seq analysis of LN biopsy samples from RM infected with WT BAC or vCD200 N.S. RRV. Variances in cellular gene expression profiles were detected between pre- and post-infection samples in all animals infected with RRV, and also between WT BAC- and vCD200 N.S.-infected RM, providing new information regarding the effects of *de novo* RRV infection, and vCD200 expression, on the cellular environment in immune tissues *in vivo*. Alterations in several genes postulated to be associated with cancer and lymphoma development were found to be differentially expressed in infected LN tissues from RRV-infected RM, including activation-induced cytidine deaminase (*AICDA*) and glypican-1 (*GPC1*), with further analysis indicating a possible connection of *GPC1* with lymphoma development. Finally, expression of vCD200 during RRV infection was also found to affect host gene expression in LN cells of infected RM, promoting the expression of thioredoxin interacting protein (*TXNIP*), a gene capable of impacting cell survival and proliferation. Overall, this data provides novel insight into cellular genes and pathways that may be important to RRV-associated disease development, and importantly, identifies genes that can be further explored in regards to their possible significance to KSHV-associated disease development in humans.

## Materials and methods

### Ethics statement

Studies were performed at the Oregon National Primate research Center (ONPRC), Beaverton, Oregon. All animal work was conducted in accordance with the recommendations of the Weatherall report, "The use of non-human primates in research." Specifically, the research was regulated by the United States Department of Agriculture for compliance with the “Animal Welfare Act” and the National Institutes of Health’s Office of Laboratory Animal Welfare for compliance with the “Guide for the Care and use of Laboratory Animals”. The ONPRC Laboratory Animal Care and Use Program is fully accredited by the American Association for Accreditation of Laboratory Animal Care (AALAC), and it has an approved Assurance (#A3304-01) for the care and use of animals on file with the Office for Protection from Research Risks at NIH. The study’s experimental protocols were approved by the Oregon Health & Science University Institutional Animal Care and Use Committee.

### Animals and animal care

Indian-origin rhesus macaques (Macaca mulatta) utilized in this study were bred and raised at the ONPRC. All animals were pair-housed in cages in environmentally controlled rooms and receive monkey chow twice daily supplemented with fresh fruit and water ad libitum. Environmental enrichment included food treats, manipulatable items in cages, human interactions with care takers and perches. All animals were observed twice daily by trained husbandry staff for activity, appetite and normal species-specific behavior. Ketamine HCl was used to induce anesthesia for all routine non-invasive clinical procedures associated with the study protocols such as blood sampling and agent administration. All surgical and invasive clinical procedures were conducted by trained personnel under the supervision of veterinarians in dedicated surgical facilities using aseptic techniques and comprehensive physiologic monitoring. Telazol^®^ was used to induce anesthesia for axillary and/or inguinal peripheral lymph node biopsies. Local analgesia at incision sites was provided by lidocaine and systemic analgesia was provided by buprenorphine. Post operatively all animals were evaluated daily for seven days to closely monitor recovery. For euthanasia macaques were sedated with ketamine HCl (15 mg/kg IM) and painlessly killed with greater than 50 mg/Kg body weight of sodium pentobarbital. Euthanasia was assured by exsanguination and bilateral pneumothorax. This method is consistent with the recommendations of the American Veterinary Medical Association’s Guidelines on Euthanasia.

### Animal infections and lymph node biopsy sample collection

Lymph node (LN) biopsy samples were obtained from a cohort of eight RM that were described previously [18]. Briefly, animals in this cohort (Cohort IV) consisted of expanded specific pathogen free (ESPF) RM that were infected intravenously with 5x10^6^ PFU of purified RRV, with 4 RM (animals 29119, 27386, 29000, and 25617) receiving WT RRV BAC-derived RRV (WT BAC), and 4 RM (animals 26430, 25662, 28902, and 28834) receiving vCD200 non-sense mutant BAC-derived RRV (vCD200 N.S.). LN biopsy samples were obtained from all animals at day 0 (d0) and day 28 (d28) post-infection (pi), from axillary (AX) and inguinal (ING) lymph nodes, respectively. At the time of LN biopsy collection, total LN cells were isolated from homogenized whole LN tissue samples, frozen in RPMI containing 10% fetal bovine serum (FBS) and 10% dimethyl sulfoxide (DMSO), and stored in liquid nitrogen until time of RNA isolation.

After the establishment of a long term latent/persistent RRV infection, cohort IV RM were infected with 5ng of SIV_mac239_ p27 antigen-containing cell-free supernatants to induce immunodeficiency. In addition, treatments were also later performed in an effort to further promote disease development in conjunction with SIV infection. Specifically, animals 27386 and 28902 were subjected to natural killer (NK) cell depletion through the administration of anti-rhesus IL-15 depleting antibody (M111R1, NIH Nonhuman Primate Reagent Resource) at days -42, -28, and -14 pre-SIV infection, the day of SIV infection, and at days 14, 28, and 42 post-SIV infection (10 mg/kg per dose), while animals 29119 and 26430 received anti-CD8α depleting antibody (cM-T807, NIH Nonhuman Primate Reagent Resource) on the day of SIV infection, and days 3 and 7 post-SIV infection (10 mg/kg per dose), to deplete both CD8+ T cells and NK cells. Finally, anti-rhesus CD8β depleting antibody (CD8beta255R1, NIH Nonhuman Primate Reagent Resource) was utilized to specifically deplete CD8+ T cells in animals 25617, 28834, 29000, and 25662, and was administered at day -71 pre-SIV infection (animals 25617 and 28834) or day 162 post-SIV infection (animals 29000 and 25662) at a dose of 50mg/kg.

### Lymph node biopsy RNA isolation and RNA-seq library preparation

RNA was isolated from total cells derived from LN tissue samples collected at d0 and d28 pi using a Quick-RNA Miniprep kit and an RNA Clean and Concentrator Kit (Zymo Research, Irvine, CA). The number of frozen cells available for RNA isolation from each animal and timepoint was variable, ranging from approximately 4x10^6^ to 9x10^6^ cells.

RNA-seq libraries were prepared by the OHSU Massively Parallel Shared Sequencing Resource (OHSU MPSSR), using total RNA and a TruSeq RNA-seq kit (Illumina, San Diego, CA). Briefly, poly(A)+ RNA was isolated using oligo-dT coated magnetic beads, chemically fragmented, and converted to double stranded cDNA using random priming. The resultant double stranded DNA ends were blunted, a single “A” nucleotide was added to the 3’ end of each strand, and standard barcoded Illumina adaptors were ligated to the cDNA. The resulting libraries were cleaned using AMPure beads (Agencourt) and amplified by a limited number of rounds of PCR. The final concentration of each library was determined by real time PCR (Kapa Biosystems). Samples were combined for multiplexing and the concentration of the final mix determined by real-time PCR and diluted to the appropriate concentration. The flow cells were run on a HiSeq 2500 using a single read 100 cycle protocol, and base call files were converted to standard fastq format using Bcl2Fastq (Illumina).

### RNA-seq data analysis

The quality of fastq files was assessed using FastQC (v0.11.3) [20] combined with MultiQC (http://multiqc.info). Fastq files were imported into PRIMe-Seq, and Trimmomatic [21] was used to trim the reads. Low quality bases at the beginning and end of each read were trimmed as well as any remaining Illumina adapters. Reads with less than 25 bases remaining were discarded. Version 7 of the RM genome (http://www.unmc.edu/rhesusgenechip/) was used along with its corresponding annotation (v7.6.8) [22], and the program STAR (v020201) [23] was used to align the reads to the genome. Counts for all samples were merged into one file, and low count genes were filtered out prior to normalization. Genes with at least 5 counts in at least 3 samples were retained for further analysis. The counts were imported into Bioconductor’s (v3.1) DESeq2 [24] for differential gene expression analysis using the DESeq2 rlog normalization approach. For RRV viral gene counts, the annotated RRV_17577_ genome (accession number AF083501.3) was used to align sequence reads.

Custom statistical modeling was performed by The Oregon National Primate Research Center (ONPRC) Bioinformatics & Biostatistics Core (BBC) to evaluate the effects of RRV infection and vCD200 expression over longitudinal changes. Mixed linear models (gene-by-gene repeated measures ANOVA) were used with mutation status (WT BAC versus vCD200 N.S.) as a between group factor and time point (d0 versus d28 pi) as a within group factor. Pairwise comparisons under repeated measures ANOVA were performed, and compound symmetry (CS) was used as correlation within each animal. False Discovery Rate (FDR) adjustment was used to control for multiple comparisons. DESeq2’s differential expression analysis method was also used to confirm results from custom modeling.

Volcano plots and lists of significantly altered genes were generated using the Shiny Transcriptome Analysis Resource Tool (START) (https://kcvi.shinyapps.io/START/). The online tool Venny (http://bioinfogp.cnb.csic.es/tools/venny/) was utilized to perform cross comparisons of infection groups and generate accompanying Venn diagrams. For cross comparison analyses, genes from WT BAC and vCD200 N.S. infection groups displaying a +/- 1.45-fold change or greater in expression from day 0 to day 28 post-infection (after rounding to 3 significant digits), and with an accompanying FDR p value of 0.05 or less, were utilized.

### qRT-PCR analysis of cellular gene expression

Primer pairs for qRT-PCR analysis of cellular RM genes were designed using NCBI Primer-BLAST (https://www.ncbi.nlm.nih.gov/tools/primer-blast/) with parameters that required each PCR product to span at least one intron, or span an exon-exon junction, such that only RNA transcripts from the associated genes were capable of being amplified from RNA samples. The RM genes analyzed by qRT-PCR, their corresponding NCBI reference sequence, and designed primer pairs utilized for each gene are as follows: *AICDA* (XM_015151031.1; Forward—GTCGGCATGAGACCTACCTG, Reverse—CACGTGACAGCCGGACTTAT), *GPC1* (NM_001266539.1; Forward—GCCAGATCTACGGAGCCAAG, Reverse—GCAGATGCTCACCCGAGAT), *CX3CR1* (NM_001265871.1; Forward—ACGCCAGGCTGTCACCAT, Reverse—ACAAATTTCCCACCAGGCCA), *RasD1* (NM_001266563.1; Forward—TCCATCCTCACAGGAGACGTT, Reverse—TTGGTGTCGAGGATCTGCTG), *TXNIP* (NM_001257935.1; Forward—GAAGACCAGCCAACAGGTGA, Reverse—GGGAAGCTCAAAGCCGAACT), *MMP-12* (XM_001098589.3; Forward—TTTTTGCACGTGGAGCTCAT, Reverse—GCTCATGAACAGCAACGAGG), *AURKA* (NC_027902.1; Forward—CAGGCATCATGGACCGATCT, Reverse—TTTGAAGGACACAGGACCCG), *CCR5* (NC_027894.; Forward–GAGCTGAGACATCCGTTCCC, Reverse–ACACCAGTGAGTAGAGCGGA), *CCL5* (XM_015119022.1; Forward–TGACTCACCTCTCCCACAGG, Reverse–TACTCCTTGATGTGGGCACG), *LAG3* (NC_027903.1; Forward–CTGGGACCTACATCTGCCAT, Reverse—CTGGAGTCACCTCACAAAGCA), *NUGGC* (XM_015145044.1; Forward–GCATGTTTGAAAGGGCCCAG, Reverse–TTCACTCCCGACATCTGCAA).

LN RNA samples for each animal and timepoint were converted to cDNA using SuperScript III First-Strand Synthesis SuperMix (Invitrogen) and qPCR was performed with Power SYBR Green PCR Master Mix using a StepOnePlus Real-Time PCR system (Applied Biosystems). Individual samples were analyzed in duplicate reactions using primers specific for each gene of interest, as well as duplicate reactions utilizing rhesus macaque *GAPDH* primers, and copy numbers were determined using the relative standard curve method. Average values obtained for each gene were normalized to average *GAPDH* values, and fold change values of increased or decreased expression were determined by calculating the ratio of normalized expression levels at d28 pi versus d0, or d0 versus d28 pi, respectively.

### Cell sorting for RNA isolation

4x10^6^ to12x10^6^ archived frozen LN biopsy cells were thawed, counted, and subjected to sequential sorting using magnetic beads specific for non-human primate CD20, CD3, and CD14 (Miltenyi Biotech, Bergisch Gladbach, Germany). Analysis of representative samples by flow cytometry indicates >97% purity of each population after sorting. Each sorted population, as well as remaining unsorted cell populations, were resuspended in RNA lysis solution and total RNA was isolated using a Quick-RNA Miniprep kit and an RNA Clean and Concentrator Kit (Zymo Research).

### Tissue staining

LN biopsy tissue was collected from infected RM at d0 and d28 pi, and either placed in neutral-buffered formalin or neutral-buffered 4% paraformaldehyde for paraffin embedding. Sections from the LN were cut at 4 μm, deparaffinized and hydrated. After appropriate blocking with 5% normal goat serum and 5% bovine serum albumin and quenching, sections were incubated with for immunostaining with primary antibodies specific for AICDA (rabbit anti-AICDA-1; Novus biologicals; 1:1000), Glypican-1 (rabbit anti-Glypican-1; Novus biologicals 1:250) T cells (rabbit anti-human CD3, Dako, Carpinteria, CA, 1:200), B cells (mouse anti-CD20, clone L26, Dako, 1:200). For immunofluorescence detection, secondary biotinylated goat anti-mouse (Vector Laboratories) or goat anti-rabbit IgG (H + L) (Vector Laboratories) were used, followed by treatment with an Elite ABC kit (Vector Laboratories), and then further stained with streptavidin Alexa 488 (Molecular Probes, Eugene, OR) and streptavidin Alexa 594 (Molecular Probes), respectively, to visualize the antigens of interest. The sections were counterstained with 4′,6-diamino-2-phenylindole dihydrochloride (DAPI) (1:5000) and covered with Prolong gold anti-fade medium (Invitrogen, Carlsbad, CA) or Omnimount (National Diagnostics, Atlanta, Georgia). Sections were examined using a Zeiss Axio Imager M1 microscope (Carl Zeiss, Thornwood, NY) using Plan NeoFluar objective lenses (2.5 ×/0.5 NA and 40 ×/0.75 NA). Optical images were obtained with standard conditions of illumination and exposure by scanning on an Olympus VS110 slide scanner on a 20x objective (Olympus). Sections were routinely stained with isotype control monoclonal antibodies and the appropriate secondary antibodies, or secondary antibodies alone as negative controls.

### Flow cytometry analysis

Total cells isolated from d0 and d28 pi LN biopsy samples and periorbital lymphoma tissue from animal 29119 were stained with antibodies directed against surface molecules CD20 (Biolegend, San Diego, CA), CD3 (BD Pharmigen, San Diego, CA), CD14 (Biolegend), and stained with Glypican 1 antibody (Novus Biologicals, Centennial, CO) conjugated to APC. Glypican 1 antibody was conjugated to APC using an APC conjugation kit (Abcam, Cambridge, UK). Cells were first stained with surface antibodies, fixed using FACS Lyse solution (Biolegend), washed with Permeabilization Wash Buffer (Biolegend), and then stained with conjugated Glypican 1 antibody. Cell were first gated on surface markers, then gated on Glypican 1, to determine percentage of Glypican 1+ cells in each subset. All samples were acquired using an LSRII instrument (BD Bioscience, San Jose CA), and data was analyzed using FlowJo software (TreeStar, Ashland, OR).

### Statistical analysis

Unpaired two-tailed *t*-tests were performed using Prism software (version 8.1.2).

## Results

### Animal samples and RNA-seq analysis

In a previous *in vivo* infection study, 4 independent cohorts of naïve RM were infected with a BAC-derived vCD200 mutant RRV (vCD200 N.S.) (n = 12), and were directly compared to control RM infected with WT BAC-derived RRV (WT BAC) (n = 11) [18]. As is typically observed after RRV infection, all RM displayed measurable viral loads in whole blood (WB) from days 14 to 42 pi, with peak viral loads occurring between d21 to d28 pi. However, on average, vCD200 N.S.-infected RM displayed higher average peak viral loads at d21 and d28 pi when compared to WT BAC-infected RM. Further, for one cohort in this study (cohort IV; WT BAC *n* = 4, vCD200 N.S. *n* = 4), LN biopsy samples were obtained at defined times pi, and examination of viral loads in LN biopsy samples from these animals revealed that vCD200 N.S.-infected RM displayed higher average peak viral loads in LN tissue at d21 and d28 pi, when compared to WT BAC-infected RM, with a statistically significant difference occurring between the two infection groups at d28 pi [18].

In an attempt to better understand the observed difference in viral load levels due to vCD200 expression, and to simultaneously assess the effects of *de novo* RRV infection on host cell gene expression patterns in LN tissue of infected RM, RNA-seq analysis was performed on RNA isolated from LN biopsy samples from all eight animals in cohort IV. Specifically, given that a statistical difference in viral DNA loads was detected in LN samples between infection groups at d28 pi, RNA was isolated from these samples, while RNA isolated from d0 LN biopsies collected just prior to infection served as a baseline control (Table 1). Due to limitations in LN biopsy tissue sample sizes, and the relatively low abundance of cells obtained from these samples, cells were not sorted into individual cell types prior to RNA purification and library preparation. Therefore, the resulting samples obtained largely represent RNA derived from total immune cells in LN, but also contain RNA from LN tissue stromal cells. After RNA purification and library preparation, RNA-seq analysis was performed on d0 and d28 pi samples from all eight animals. This analysis resulted in the identification of over 13,000 cellular genes with normalized counts, as well as the detection of transcripts from RRV genes.

**Table 1:** Animal infections and lymph node biopsies.

		Lymph Node Biopsy^b^	
Infection Group^a^	Animal No.	Day 0	Day 28
WT BACWT BAC	29119	AX LN	ING LN
	27386	AX LN	ING LN
	29000	AX LN	ING LN
	25617	AX LN	ING LN
vCD200 N.S.	26430	AX LN	ING LN
	25662	AX LN	ING LN
	28902	AX LN	ING LN
	28834	AX LN	ING LN

^a^ Each animal received 5x10^6^ PFU of WT or vCD200 N.S. BAC-derived RRV intravenously

^b^ Day post-infection of lymph node biopsy samples; AX LN (axillary lymph node), ING LN (inguinal lymph node)

**Fig 1:**
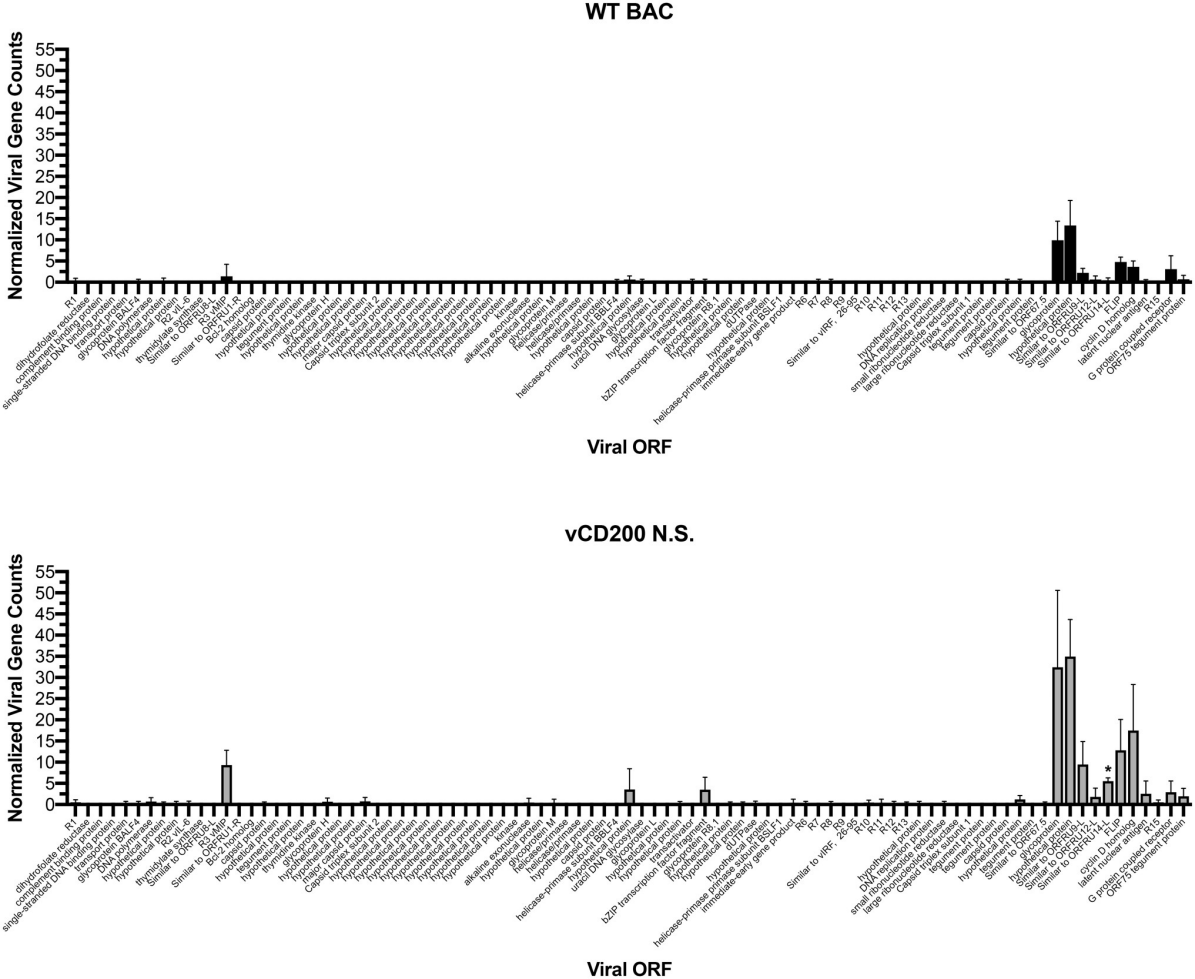
Viral gene expression in LN biopsy samples of RRV-infected RM. RNA-seq data obtained from d28 pi LN biopsy samples of WT BAC and vCD200 N.S. infection groups was analyzed for RRV transcript expression. Values presented are the averages of normalized viral gene counts for all animals within each infection group (*n* = 4), with error bars indicating standard deviation. RRV gene names are listed on the X axis as organized from 5′ to 3′ in the RRV_17577_ genome. ORFRU14-L was the only viral gene found to display a significant difference in expression levels between infection groups (*, *p* = 0.0023, unpaired *t*-test).

Data from individual animals was compiled based on infection group and timepoints, and then subjected to analysis using custom statistical modeling. Initially, RRV gene expression patterns were assessed, confirming the presence of RRV transcripts only in d28 pi samples (Fig 1). Interestingly, slightly higher expression levels of viral genes were detected in LN biopsy samples of vCD200 N.S.-infected RM, a finding which is likely attributable to the fact that on average, more virus was present in LN tissue from these animals [18]. Despite this, comparison of viral gene expression levels between infection groups only identified one gene with a statistically significant difference in expression, ORFRU14-L, a gene with undescribed function, which displayed average normalized counts of 0.48 and 5.5 in d28 LN biopsy samples of WT BAC and vCD200 N.S.-infected RM, respectively (*p* = 0.0023, unpaired *t*-test). In general, the patterns of viral gene expression indicate that the majority of RRV genes expressed in d28 LN are those located near the right end of the viral genome, a region that contains the latency-associated genes ORF 71, 72, and 73, which encode the viral FLIP homolog (vFLIP), cyclin D homolog (vCyc), and latency-associated nuclear antigen (LANA), respectively (Fig 1). RRV ORF R15 encodes vCD200, and is transcribed as part of a lytic bi-cistronic message that also contains the downstream ORF74, which encodes the viral G protein-coupled receptor (vGPCR) [25]. Though transcripts from R15-ORF74 and other known lytic genes such as R3 (vMIP) remain detectable, the predominance of gene expression in the latency region suggests that viral gene expression patterns are trending towards latency in LN from infected RM by d28. This timepoint coincides closely with the onset of decreased peak viral loads in all infected RM, and the eventual establishment of a latent/persistent infection by approximately day 56 pi [18]. Overall, however, the general viral gene expression profiles are similar in both WT BAC and vCD200 N.S.-infected RM, indicating that the absence of vCD200 does not drastically alter RRV gene expression in LN tissue.

### RRV infection induces changes in cellular gene expression profiles in LN tissue

**Fig 2:**
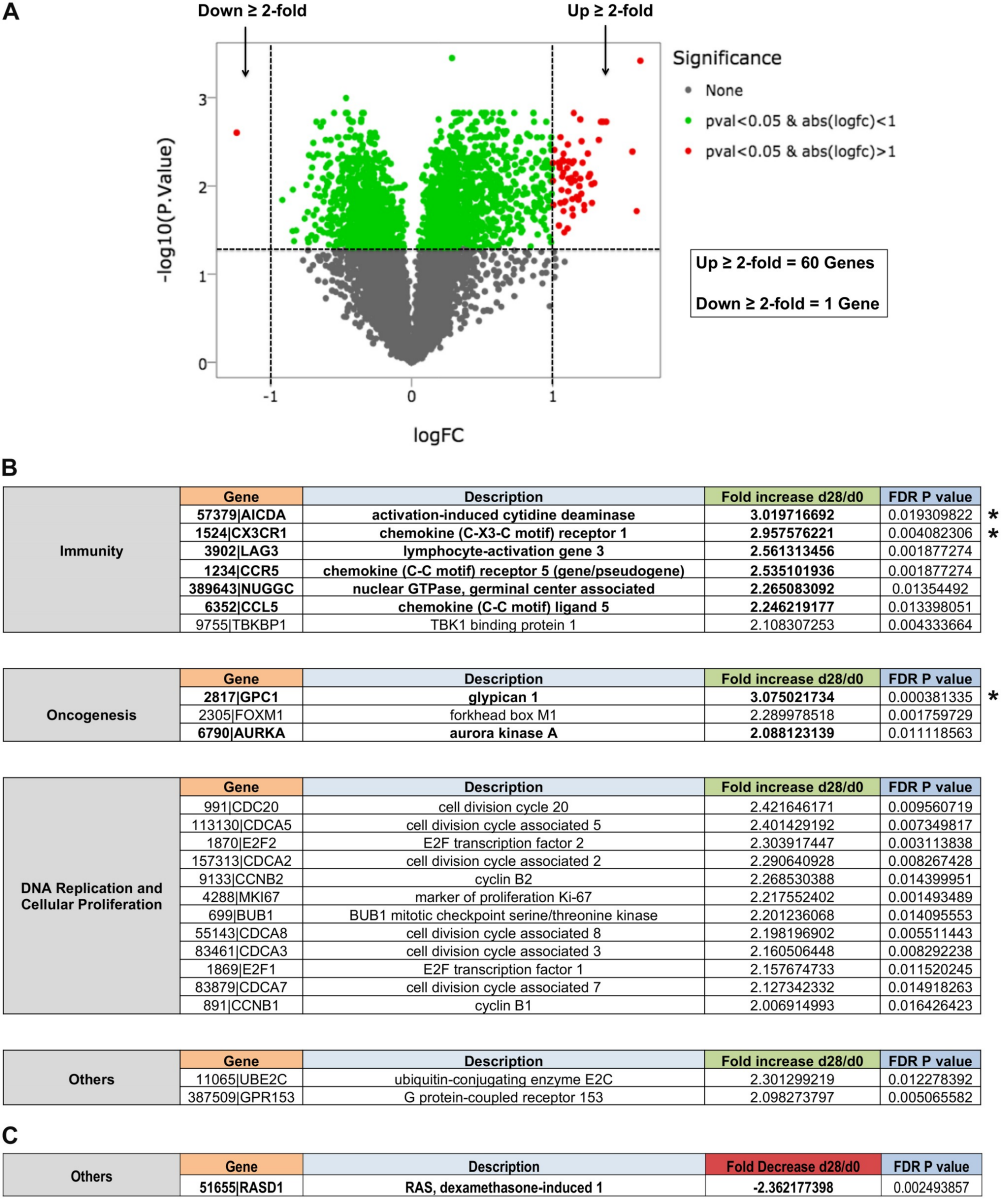
Effects of RRV infection on cellular gene expression profiles in LN tissue. (A) Volcano plot displaying the log_2_ fold-change in expression from d0 to d28 pi, and the associated -log_10_ FDR *p* value, for all cellular genes detected by RNA-seq analysis in LN biopsy samples of all 8 RRV-infected RM combined. Only those genes that displayed a ≥ 2-fold change in expression from d0 to d28 pi, with an associated FDR *p* value of <0.05, were considered for further analysis. Using these criteria, 60 genes were found to be significantly upregulated, and only 1 gene was found to be significantly downregulated after RRV infection. Upregulated (B) and downregulated (C) genes of interest were sorted into categories based on putative functionality. Asterisks indicate the 3 genes displaying the highest changes in expression overall, and genes in bold font indicate those assessed by follow-up qRT-PCR analysis.

In order to assess the overall effects of RRV infection on changes in cellular gene expression profiles in infected RM, analyses were performed to compare d0 and d28 pi expression profiles from all eight infected animals combined, regardless of the virus type utilized for infection. Using the selection criteria of a ≥ 2-fold change in expression levels from d0 to d28 pi, and an FDR adjusted *p* value of ≤ 0.05 as a cut-off value for the level of significance, genes either up- or down-regulated after RRV infection were identified. Based on these criteria, 60 genes displayed a ≥ 2-fold increase in expression from d0 to d28 pi, while only one gene displayed a >2-fold decrease in expression after RRV infection (Fig 2A). Genes of interest were next identified from this list based on their putative functional properties and potential relevance to both KSHV and RRV infection and disease development, and were then manually sorted into functional categories of immunity, oncogenesis, DNA replication and cellular proliferation, as well as other genes of interest not strictly associated with these categories (Fig 2B and 2C).

A majority of genes up-regulated after RRV infection were found to be associated with DNA replication and cellular proliferation (e.g. cyclins and cell division cycle associated proteins), which is likely attributable to the generalized increase in cellular activation due to RRV infection, and the induction of immune cell proliferation due to the development of anti-RRV immune responses. For example, Ki67, a nuclear proliferation marker whose expression was previously found to be elevated at d28 pi by flow cytometry in CD4+ and CD8+ T cells in PBMC samples from these same animals [18], was also found to be up-regulated at d28 pi in LN biopsy samples by RNA-seq analysis, suggesting that gene expression alterations observed *in vivo* can also be associated with protein level changes in RRV-infected RM.

Although elevated proliferation and cell division markers likely correlate with a general increase in replication of immune cells within LN tissue, of more specific interest was the identification of genes associated with oncogenesis and immune regulation that display elevated or decreased expression after RRV infection, since these genes are most likely to be associated with alterations that affect anti-RRV immune responses, or affect cellular pathways relevant to RRV-associated disease development. Examination of these categories identified several genes of interest, three of which had the highest changes observed overall in our analysis, with the highest change occurring for *GPC1* (fold change +3.0750), followed by *AICDA* (fold change +3.0197), and *CX3CR1* (fold change +2.9575).

**Fig 3:**
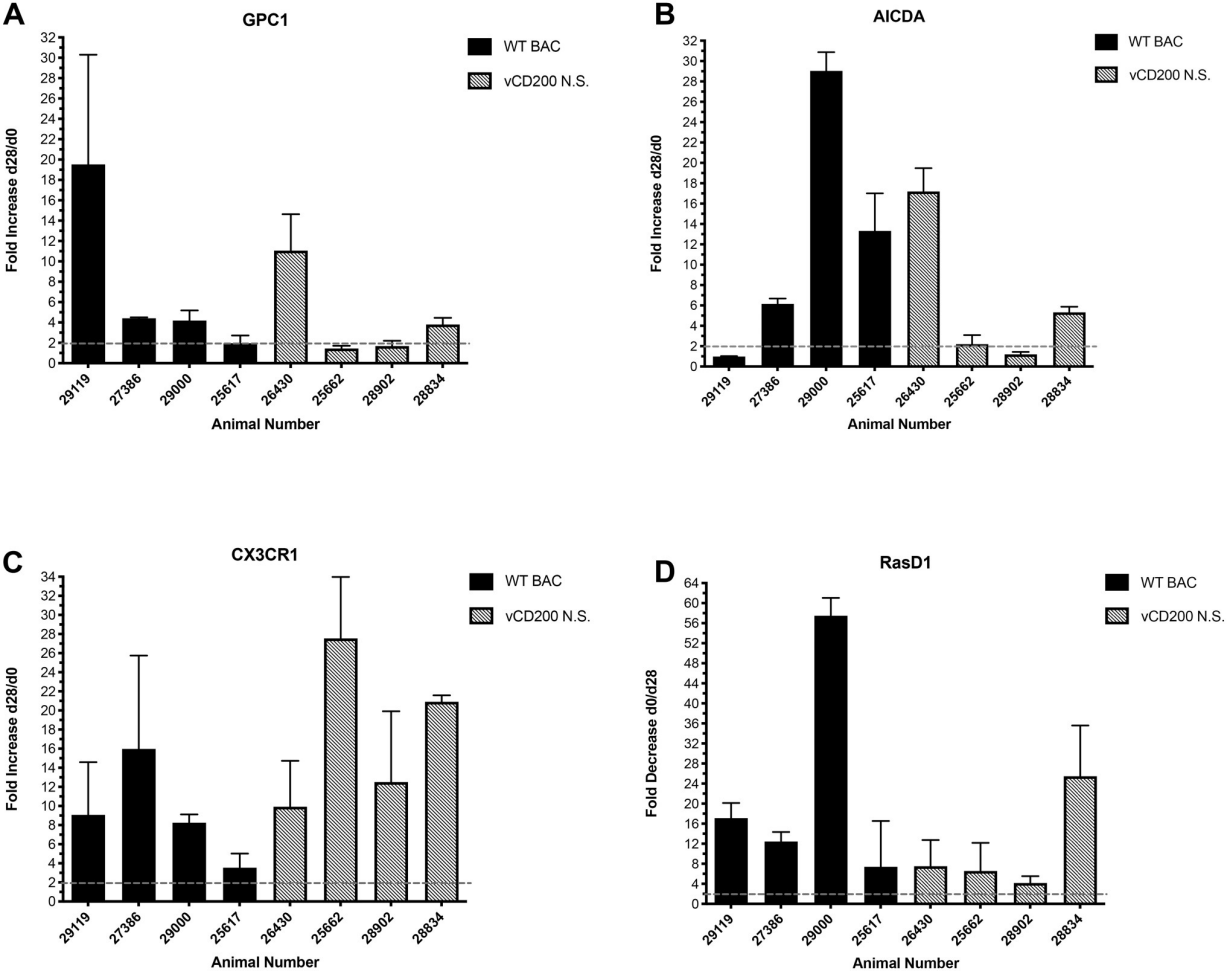
qRT-PCR analysis of LN biopsy samples from individual RM. RNA samples from d0 and d28 post-infection LN biopsies of individual RM were analyzed by qRT-PCR for the expression of *GPC1* (A), *AICDA* (B), *CX3CR1* (C), and *RasD1* (D). Data represents fold change in normalized gene expression values (gene copy number/*GAPDH* copy number) at d28 post-infection compared to d0 values. Error bars denote standard deviation, and a dashed line indicates 2-fold change value. Individual animal numbers and their associated infection groups are noted.

From this initial group of genes displaying a ≥ 2-fold change at d28 pi, several genes involved in chemokine signaling (*CX3CR1*, *CCR5*, *CCL5*), antibody diversification (*AICDA*, *NUGGC*), lymphocyte signaling (*LAG3*), B cell regulation (*RasD1*), and with connections to oncogenesis and cancer (*GPC1*, *AURKA*), were chosen for follow-up qRT-PCR analysis, to confirm observations made by RNA-seq. To achieve this, primers were designed to specifically amplify transcripts of these genes, and d0 and d28 pi LN RNA samples from individual RM were tested for transcript expression levels of each gene, as well as *GAPDH* as a control for normalization (Table 2). Next, a comparison of d28 pi versus d0 normalized expression levels (gene copies/*GAPDH* copies) for each gene of interest was made to determine the overall fold-increase in expression in individual animals after RRV infection. Of the 8 RRV up-regulated genes examined, only *GPC1*, *AICDA*, and *CX3CR1* were successfully confirmed by qRT-PCR as displaying elevated expression levels in individual animals similar to those detected by RNA-seq analysis of RNA from these same tissue samples. Specifically, a ≥ 2-fold increase in transcript expression was detected in 6 of 8 RM analyzed for *GPC1* and *AICDA* expression, and all 8 RM analyzed for *CX3CR1* expression levels (Fig 3). The only gene identified that displayed a ≥ 2-fold reduction after RRV infection, *RasD1*, was found to be downregulated in all 8 RM by qRT-PCR analysis, further confirming the RNA-seq observations made for this gene (Fig 3D). Though a lack of confirmation by qRT-PCR of the remaining genes tested does not diminish observations made by RNA-seq analysis, those genes capable of being confirmed in this manner are further strengthened in their potential relevance to RRV-associated changes in cellular gene expression profiles in LN tissue of RRV-infected RM.

**Table 2:** Gene-specific primers utilized for qRT-PCR analysis.

Gene ^a^	Forward Primer (5’– 3’)	Reverse Primer (5’– 3’)
*AICDA*	GTCGGCATGAGACCTACCTG	CACGTGACAGCCGGACTTAT
*AURKA*	CAGGCATCATGGACCGATCT	TTTGAAGGACACAGGACCCG
*CCL5*	TGACTCACCTCTCCCACAGG	TACTCCTTGATGTGGGCACG
*CCR5*	GAGCTGAGACATCCGTTCCC	ACACCAGTGAGTAGAGCGGA
*CX3CR1*	ACGCCAGGCTGTCACCAT	ACAAATTTCCCACCAGGCCA
*GPC1*	GCCAGATCTACGGAGCCAAG	GCAGATGCTCACCCGAGAT
*LAG3*	CTGGGACCTACATCTGCCAT	CTGGAGTCACCTCACAAAGCA
*MMP-12*	TTTTTGCACGTGGAGCTCAT	GCTCATGAACAGCAACGAGG
*NUGGC*	GCATGTTTGAAAGGGCCCAG	TTCACTCCCGACATCTGCAA
*RasD1*	TCCATCCTCACAGGAGACGTT	TTGGTGTCGAGGATCTGCTG
*TXNIP*	GAAGACCAGCCAACAGGTGA	GGGAAGCTCAAAGCCGAACT

^a^ *Macaca mulatta* gene name

Interestingly, the 4 genes identified by RNA-seq and confirmed by qRT-PCR as being the most highly up- or down-regulated after RRV infection all possess functional characteristics of genes with the potential to be involved in immune responses against RRV, or in the development of RRV-associated malignancies. For example, *CX3CR1* is the receptor for fractalkine, and is involved in normal B cell function and immune regulation, but has also been found to be overexpressed in B cell lymphomas and B-CLL in humans [26–28], suggesting that a similar upregulation due to RRV infection may also be associated with disease development in RM. Alternatively, *RasD1*, the single gene identified as being downregulated > 2-fold after RRV infection, is a member of the Ras superfamily of G proteins, and has been implicated as a potential regulatory protein in B lymphocytes that may play a role in inhibiting B lymphocyte activity and proliferation [29]. *RasD1* has also been found to be capable of inhibiting the growth of cancer cells [30], and thus, decreased expression of *RasD1* in RM LN tissues after RRV infection could potentially promote increased B cell proliferation and the development of B cell abnormalities in RRV-infected RM.

**Fig 4:**
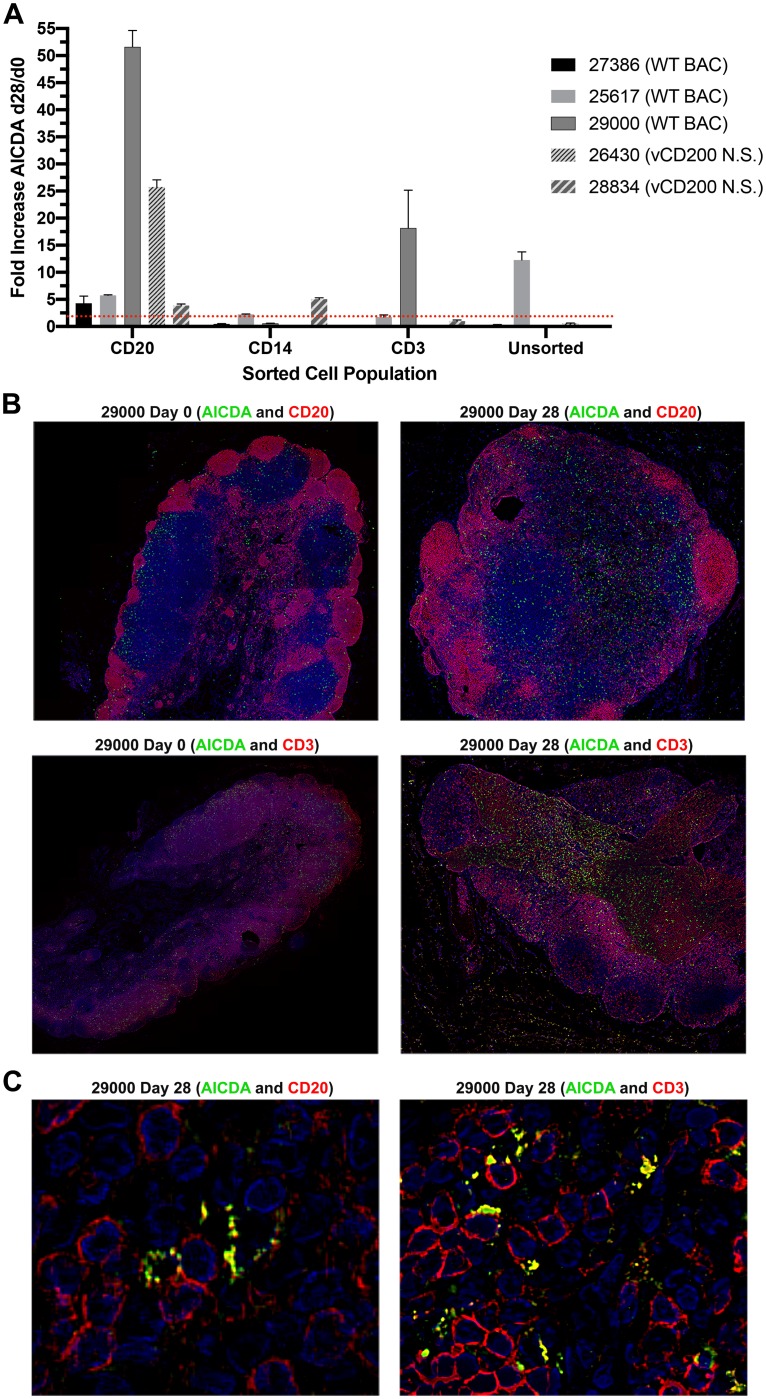
AICDA expression in sorted cell populations and tissue sections of LN biopsy samples. (A) Cells from LN biopsy samples of three WT BAC and two vCD200 N.S.-infected RM were subjected to sorting using magnetic beads to obtain purified CD20+, CD14+, and CD3+ cell populations, and RNA was isolated from each population as well as remaining unsorted cells for use in qRT-PCR using *AICDA*-specific primers. Due to lack of sufficient RNA, unsorted samples of animals 29000 and 28834, and CD14 and CD3 samples of animal 26430, were not capable of being analyzed. Data represents fold change in normalized gene expression values (gene copy number/*GAPDH* copy number) at d28 pi compared to d0 values, and error bars indicate standard deviation. (B) Tissue staining of LN biopsy samples from animal 29000. Tissue sections were stained with anti-AICDA antibody (green), anti-CD20 or anti-CD3 antibody (red), and DAPI (blue) to stain nuclei. Original magnification X20. (C) Enlarged images of d28 pi tissue sections from animal 29000 demonstrating co-localization of AICDA with CD20+ B cells (left panel) and CD3+ T cells (right panel).

Another gene confirmed to be upregulated after RRV infection, and of particular interest due to its role in B cell function, is activation-induced cytidine deaminase (*AICDA*). *AICDA* is induced in germinal center (GC) B cells upon antigen exposure, and is known as a major regulator of antibody diversification, where it is involved in both somatic hypermutation and class switching [31, 32]. Importantly, despite its normal role in development of antibody responses, the overexpression of *AICDA* also has the potential to promote off-target mutations, and has been implicated in the development of a variety of cancers, including lymphoma [33–35]. Normally, *AICDA* expression is tightly regulated, and though predominantly restricted to GC B cells, expression of this protein has also been found in some instances to occur in other cell types, where aberrant expression can lead to development of a variety of non-B cell cancers [31, 36]. In an attempt to identify cell types in which *AICDA* expression is induced in LN of RRV-infected RM, LN biopsy cells were subjected to sequential magnetic bead sorting to obtain purified CD20, CD14, and CD3 cell populations, and RNA was isolated from these subsets, as well as remaining unsorted cells, for use in qRT-PCR analysis. Due to limiting cell numbers, this analysis was only performed on sorted cells obtained from animals that displayed > 2-fold increase in the initial analysis of *AICDA* expression of total LN cells via qRT-PCR (Fig 3B), and included 3 WT RM (27386, 25617, 29000) and 2 vCD200 N.S. RM (26430 and 28834). As anticipated, the most consistent and robust changes in *AICDA* expression occurred in CD20+ B cells from these animals, with all 5 animals examined displaying a > 2-fold increase in expression, and animals 26430 and 29000 demonstrating the most dramatic changes, with 25.7-fold and 51.6-fold increases in expression from d0 to d28, respectively (Fig 4A). Interestingly, > 2-fold increases in *AICDA* expression were observed in the CD14+ subset from animals 25617 and 28834, the CD3+ subset of animal 29000, and in the unsorted subset of animal 25617, suggesting *AICDA* expression may also be induced in non-B cell populations after RRV infection in some instances. Overall, however, these findings demonstrate that *AICDA* gene expression is induced largely in B cells after RRV infection. In an attempt to examine changes AICDA protein expression in LN tissues, staining was performed on LN tissue sections of RM 29000, which displayed the highest level of induction in gene expression in total LN cells and CD20+ B cells by qRT-PCR, and for which tissue sections were available. Staining of this tissue revealed that AICDA protein expression was visibly elevated at d28 pi, when compared to d0 (Fig 4B), and although localized with B cells, AICDA was also associated with CD3+ T cells in these tissues (Fig 4C). These staining patterns parallel the RNA expression patterns observed in sorted cell subsets obtained from this same animal. Taken together, these findings confirm that RRV infection induces AICDA gene and protein expression in LN of infected RM *in vivo*, implicating the potential involvement of this gene in RRV associated disease development.

Overall, the most highly upregulated gene identified by RNA-seq analysis of LN samples from RRV-infected RM was Glypican 1 (*GPC1*) (Fig 2). GPC1 is a member of a family of cell surface proteoglycans that promote formation of ligand-receptor complexes, enhancing signaling of cell surface receptors such as fibroblast growth factor (FGF), transforming growth factor-β (TGF-β), and vascular endothelial growth factor (VEGF), the effects of which can ultimately promote cell proliferation and oncogenesis [37]. Indeed, the ability of GPC1 to modulate cellular adhesion and motility has been implicated in promoting the growth and angiogenic and metastatic potential of cancer cells in a variety of models [38]. Of further importance, GPC1 has also been found to be enriched in cellular exosomes from cancer types ranging from pancreatic to colorectal cancer [37, 39, 40], and has been investigated as a potential biomarker for the early detection of pancreatic cancer [40]. Thus, a particularly intriguing finding was the fact that the highest induction of *GPC1* gene expression was observed in animal 29119, the only animal in the entire cohort to develop lymphoma.

### Increased Glypican-1 expression is associated with lymphoma development *in vivo*

As part of our standard protocol to assess RRV-associated disease development, cohort IV animals were infected with SIV_mac239_ after establishing long-term latent/persistent RRV infections. Further, in an attempt to assess additional methods to enhance disease development, animals in this cohort were also differentially subjected to treatment with anti-IL-15 antibody to deplete NK cells (animals 27386 and 28902), anti-CD8α antibody to deplete both NK and CD8 T cells (animals 29119 and 26430), or anti-CD8β antibody to deplete only CD8 T cells (animals 25617, 28834, 25617, and 28834) (Table 3). By the end of the study period, RM 29199 was the only animal to present with RRV-associated disease, with the detection of a periorbital lymphoma at the time of necropsy. As only 1 of 8 RM in this cohort developed lymphoma, NK cell and/or CD8 T cell depletion did not specifically enhance viral-associated disease development, which typically occurs in 20–30% of RRV/SIV co-infected animals [2]. Of specific interest, however, was the fact that lymphoma development occurred in the same animal that also displayed the highest average level of induction of *GPC1* gene expression in d28 pi LN biopsy samples.

**Table 3:** Secondary treatment strategies and lymphoma development in RRV-infected RM.

		Secondary Treatments				
Animal No.	Primary RRV Infection^a^	SIV_mac239_^b^	anti-IL-15^c^	anti-CD8α^d^	anti-CD8β^e^	Lymphoma Development
29000	WT BAC	+	-	-	+	-
25662	vCD200 N.S.	+	-	-	+	-
29119	WT BAC	+	-	+	-	+
26430	vCD200 N.S.	+	-	+	-	-
27386	WT BAC	+	+	-	-	-
28902	vCD200 N.S.	+	+	-	-	-
25617	WT BAC	+	-	-	+	-
28834	vCD200 N.S.	+	-	-	+	-

^a^ Intravenous inoculation with 5x10^6^ PFU of WT or vCD200 N.S. BAC-derived RRV.

^b^ Intravenous inoculation with SIV_mac239_ (5ng per animal)

^c^ anti-rhesus IL-15 depleting antibody was administered in 7 separate doses of 10 mg/kg of body weight, on days -42, -28, -14, 0, 14, 28, and 42, relative to SIV infection date.

^d^ anti-rhesus CD8α depleting antibody was administered in 3 separate doses of 10mg/kg of body weight, on days 0, 3, and 7, relative to SIV infection date.

^e^ anti-rhesus CD8β depleting antibody was administered in a single dose of 50mg/kg of body weight at day 162 post-SIV infection (animals 29000 and 25662) or day 71 pre-SIV infection (animals 25617 and 28834).

**Fig 5:**
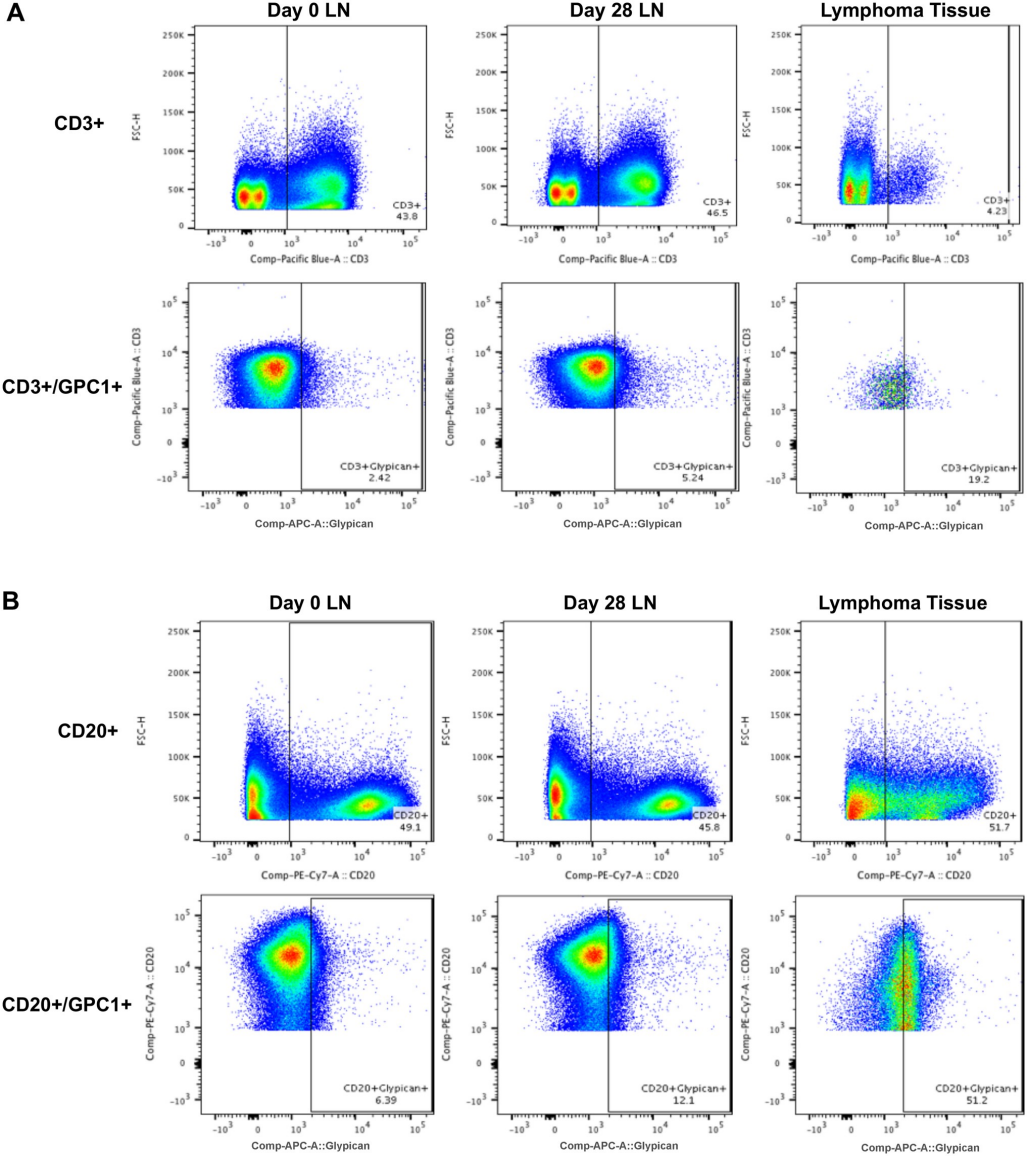
Flow cytometry analysis of Glypican-1 expression in LN biopsy and lymphoma tissues. Total cells derived from d0 and d28 pi LN biopsy and lymphoma tissue samples from animal 29119 were stained with antibodies directed against Glypican-1 (GPC1), CD20, CD3, and CD14, and analyzed by flow cytometry. Data was gated to determine the percentage of (A) CD3+ and CD3+/GPC1+ or (B) CD20+ and CD20+/GPC1+ cells in each sample. CD14+ cells represented < 1% of all cells in lymphoma and LN tissue samples.

To further explore the possible connection of *GPC1* with lymphoma development, and to assess whether changes in GPC1 protein levels could be detected in both LN and lymphoma tissue from an RRV-infected RM, cells from LN biopsies and lymphoma tissue from animal 29119 were analyzed by flow cytometry. Specifically, cells isolated from d0 and d28 pi LN biopsy and lymphoma tissue were stained with anti-GPC1 antibody, in conjunction with antibodies directed against CD20, CD3, and CD14, to determine the levels of GPC1+ cell subsets in these tissues (Fig 5). In LN samples from RM 29119, 43.8% of d0 and 46.5% of d28 pi cells were CD3+ T cells (Fig 5A), while 49.1% of d0 and 45.8% of d28 pi cells were CD20+ B cells (Fig 5B). However, in lymphoma tissue from this same animal, only 4.23% of cells stained CD3+, while 51.7% of all cells stained CD20+, indicating the predominance of B cells in lymphoma tissue compared to normal LN. Less than 1% of all cells were CD14+ in lymphoma and LN samples. Analysis of GPC1 staining of LN and lymphoma cells indicated that total GPC1 protein levels are increased in CD20+ B cells when comparing d28 pi (12.1%) to d0 LN samples (6.39%), while a slight increase in GPC1 staining was also observed from d0 to d28 pi in CD3+ cells from LN samples (5.24% versus 2.42%). In general, this increase in GPC1 protein levels in CD20+ and CD3+ cells in d28 pi LN samples mirrors observations demonstrated by RNA-seq and qRT-PCR analysis, suggesting that increased *GPC1* gene expression due to RRV infection also results in higher levels of GPC1 protein expression in LN cells. More interestingly, however, was the robust increase in GPC1 protein levels observed in lymphoma tissue B cells from this animal compared to levels detected in B cells from d0 and d28 pi LN samples, with 51.2% of lymphoma B cells staining positive for GPC1 expression, a 44.8% and 39.1% increase from levels detected in d0 and d28 pi LN B cells, respectively (Fig 5B). Although other factors are likely to have contributed to the eventual development of lymphoma in this animal, the elevation in GPC1 protein levels in CD20+ cells from LN after RRV infection at d28, and the high level detected in lymphoma-derived B cells, strongly suggests that *GPC1* induction has the potential to be directly associated with development of cancers in RRV-infected RM.

### Effects of vCD200 on cellular gene expression profiles

**Fig 6:**
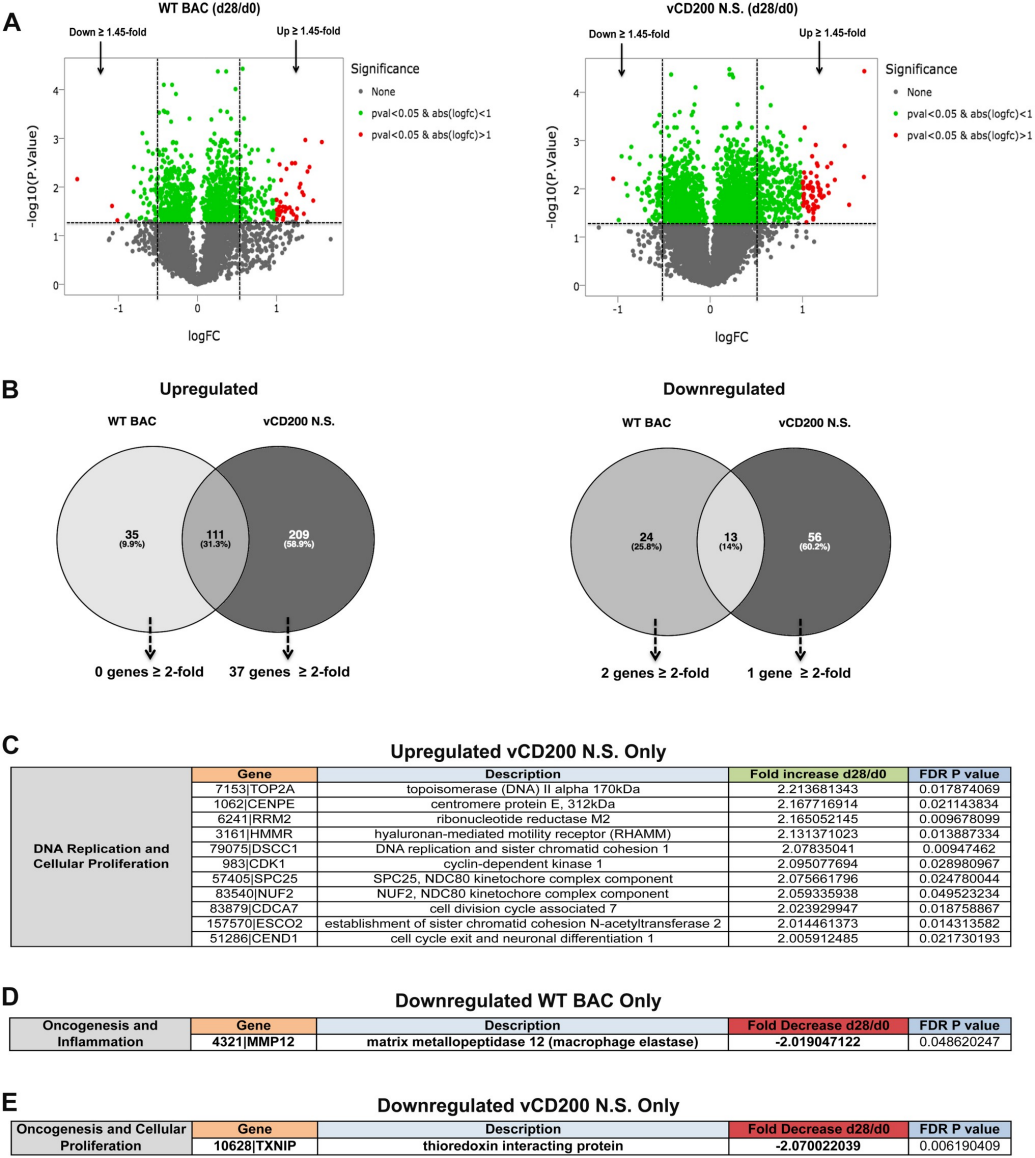
Cross comparison of cellular gene expression between WT BAC and vCD200 N.S. infection groups. (A) Volcano plot displaying the log_2_ fold-change in expression from d0 to d28 pi, and the associated -log_10_ FDR *p* value, of all cellular genes identified by RNA-seq in WT BAC and vCD200 N.S. infection groups. (B) Venn diagrams comparing genes upregulated or downregulated in WT BAC or vCD200 N.S. infection groups. Only genes displaying a ≥1.45-fold change in expression with an accompanying FDR *p* value of < 0.05 were used for cross comparison analysis. The number of genes unique to each group displaying a ≥ 2-fold change in expression are indicated. Identified genes of interest from cross comparison analysis that display specific ≥ 2-fold upregulation in vCD200 N.S. infection group (C), ≥ 2-fold downregulation in WT BAC infection group (D), or ≥ 2-fold downregulation in vCD200 N.S. infection group (E). Functional categories of genes are indicated, and genes highlighted in bold were assessed by follow-up qRT-PCR analysis.

Given that RRV vCD200 has inhibitory effects on host immune responses, and negatively affects RRV viral loads *in vivo*, this molecule is likely to alter host gene expression patterns during infection. In order to assess the contributions of RRV vCD200 to the modulation of host gene expression, and to determine if any of these changes may be associated with differences in viral loads observed between WT BAC and vCD200 N.S. viruses in LN tissue of RRV-infected RM, RNA-seq data from both infection groups was subjected to a cross comparison. Specifically, RNA-seq gene expression data for d0 and d28 pi samples was compiled separately for WT BAC (*n* = 4) and vCD200 N.S. (*n* = 4) infection groups, and a cross comparison between groups was then performed to identify genes differentially regulated during infection with each virus. When compiling data for individual infection groups, a less stringent cutoff value of ≥ 1.45-fold was utilized in order to include genes that might display fold change expression values slightly below 2 in initial cross comparisons, while the significance cut-off was maintained at an FDR *p* value of 0.05 (Fig 6A). Next, a cross comparison of all genes upregulated or downregulated ≥1.45-fold in each infection group was performed (Fig 6B), and the resulting lists of genes found to be altered only in specific infection groups were then manually examined for those displaying a ≥ 2-fold change in expression.

Examination of genes that displayed ≥1.45-fold increase in expression in individual infection groups from d0 to d28 pi revealed that the largest number of changes occurred in vCD200 N.S.-infected RM (209 genes, 58.9%), while a smaller number of genes displayed increases in expression in RM infected with WT BAC (35 genes, 9.9%). None of the 35 genes specifically increased in WT BAC-infected animals displayed an induction ≥ 2-fold, and were not examined further, while 37 of the 209 vCD200 N.S.-specific genes were upregulated ≥ 2-fold at day 28. Further analysis revealed that 3 of the 37 genes meeting criteria for upregulation in the vCD200 N.S. infection group, specifically *AICDA*, aurora kinase B (*AURKB*), and *NUGGC*, were actually similarly elevated in the WT BAC infection group at d28 pi, having only been excluded from cross comparison analysis due to the possession of FDR *p* values in this group that were slightly above the cutoff utilized for level of significance (*AICDA p* = 0.118, *AURKB p* = 0.0772, *NUGGC p* = 0.0522). In addition, *AICDA* and *NUGGC* had previously been identified as displaying significant ≥ 2-fold increases in expression when comparing d28 pi to d0 LN samples from both infection groups combined (Fig 2). Thus, overall a total of 34 genes were confirmed as being specifically and significantly upregulated in the vCD200 N.S. infection group at d28 pi. Examination of these 34 genes revealed that none possess predicted functional properties with apparent connections to immune regulation or RRV pathogenesis, with the majority being associated with cellular proliferation and DNA replication (Fig 6C). While the increase in expression of cellular proliferation and DNA replication-associated genes in vCD200 N.S. infection group at d28 pi may simply be attributable to the elevated levels of viral replication observed in vCD200 N.S.-infected animals at this timepoint, the inhibition of their expression in WT BAC-infected RM due to vCD200 expression is also a possibility, although the specific implication of particular gene(s) from this list in directly regulating RRV replication is not readily apparent.

Genes downregulated in individual infection groups from d0 to d28 pi were less in number, with 24 displaying ≥1.45-fold decrease in the WT BAC infection group, and 56 displaying ≥1.45-fold decrease in the vCD200 N.S. infection group. Genes displaying a ≥ 2-fold decrease in either group were further limited, with only 2 genes in the WT BAC infection group, coiled-coil domain containing 152 (*CCDC152*) and matrix metallopeptidase 12 (*MMP12*) (Fig 6D), and one gene in the vCD200 N.S. infection group, thioredoxin interacting protein (*TXNIP*) (Fig 6E), demonstrating a ≥ 2-fold decrease in expression from d0 to d28 pi. The lack of variable expression of genes with ≥ 2-fold expression between infection groups is likely due to more subtle effects of vCD200-CD200R signaling on gene expression patterns overall, and though less stringent selection criteria could suggest alterations in a larger number of genes, we chose to focus only on those with more detectable and robust ≥ 2-fold changes in expression. Of the 3 differentially downregulated genes identified, only TXNIP appears to have potential functional properties that could directly influence viral replication, and result in the observed phenotypic difference in viral loads measured between vCD200 N.S. and WT BAC viruses *in vivo*.

TXNIP is a widely expressed cellular protein that is a negative regulator of thioredoxin (Trx), and directly interacts with and inhibits Trx function [41]. The Trx system is a cellular thiol-reducing antioxidant system that is involved in the regulation and maintenance of a reducing environment and inhibition of reactive oxygen species (ROS) production, protecting cells from damage due to oxidative stress. However, in addition to its role in Trx regulation, TXNIP has also been found to be a multifunctional protein that can affect a wide variety of cellular processes, and whose expression has been shown to promote apoptosis, NLRP3 inflammasome activation, and inhibit cellular proliferation [41–47]. In addition, TXNIP expression has also been found to be affected by infection with several viruses, including human T lymphotropic virus type-I (HTLV-I), hepatitis B virus (HBV), hepatitis C virus (HCV), as well as herpesviruses cytomegalovirus (CMV), Epstein-Barr virus (EBV), and KSHV [48–54]. In some instances, dysregulation of TXNIP has been suggested to play a direct role in viral oncogenesis [52, 53], and to affect virus replication [54].

**Fig 7:**
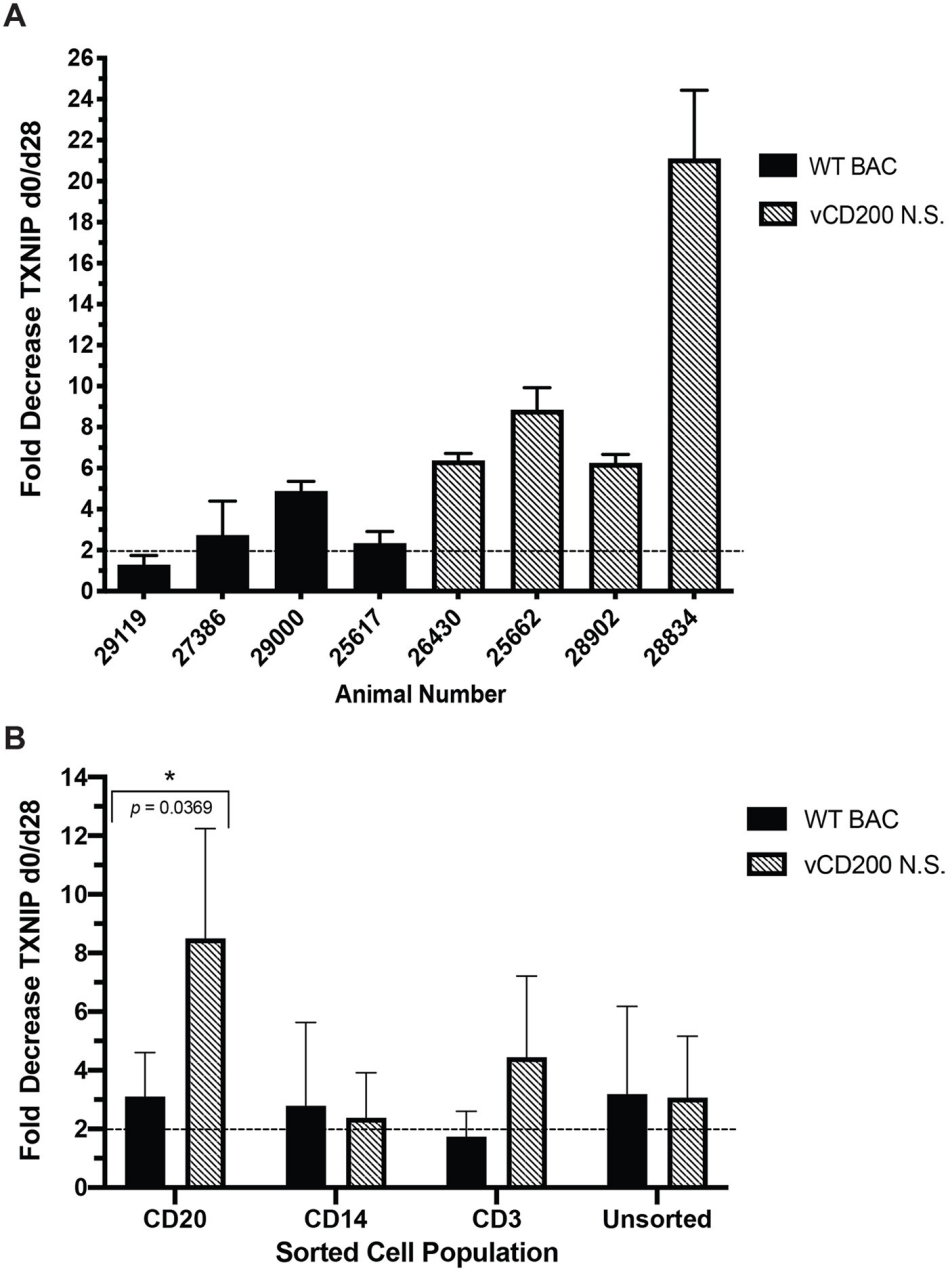
TXNIP gene expression in LN of individual RM and sorted cell populations from WT BAC and vCD200 N.S. infection groups. (A) RNA samples from d0 and d28 pi LN biopsy cells of individual RM were analyzed for expression levels of *TXNIP* by qRT-PCR. Data represents the fold change decrease in normalized gene expression values (gene copy number/*GAPDH* copy number) from d0 to d28 pi, and individual animals and infection groups are noted. Error bars denote standard deviation, and a dashed line indicates 2-fold change value. (B) Cells from LN biopsy samples of individual animals from WT BAC (animals 29119, 27386, 29000, and 25617) and vCD200 N.S. (animals 26430, 25662, 28902, and 28834) infection groups were subjected to sorting using magnetic beads to obtain purified CD20+, CD14+, and CD3+ cell populations, and RNA was isolated from each population as well as remaining unsorted cells for use in qRT-PCR using *TXNIP*-specific primers. The average fold-change decrease in normalized gene expression values (gene copy number/*GAPDH* copy number) from d0 to d28 pi for each infection group is shown. Error bars denote standard deviation, and a dashed line indicates 2-fold change value. The difference in decreased *TXNIP* expression in CD20+ cells was found to be significantly different between WT BAC and vCD200 N.S. infection groups by unpaired *t*-test (*, *p* = 0.0369).

*TXNIP* displayed a significant 2.07-fold decrease in expression levels at d28 pi by RNA-seq in vCD200 N.S. RM, but was not similarly downregulated in WT BAC-infected RM (1.27-fold decrease, FDR *p* = 0.437), excluding it from cross comparison analysis (Fig 6B). To further confirm the variance in downregulation of TXNIP between virus infection groups, RNA from individual RM was subjected to qRT-PCR analysis using primers specific for RM *TXNIP* (Fig 7A). Using this approach, a statistically significant difference in the average fold-decrease of *TXNIP* expression was not identified between infection groups, however, this analysis revealed that all four vCD200 N.S.-infected RM displayed 6.3-fold or higher downregulation of *TXNIP* expression at d28, while WT BAC-infected RM displayed much lower decreases in *TXNIP* expression, ranging from 1.3-fold to 4.9-fold. Given the roles of TXNIP in preventing cell proliferation, promoting apoptosis, and inducing inflammasome activation, it is plausible that elevated TXNIP levels in infected cells in LN tissue would negatively affect RRV replication. This finding suggests a possible mechanism that may help explain why vCD200 N.S.-infected RM, which display lower levels of *TXNIP* expression, possess higher viral loads in LN tissues than those observed in WT BAC-infected RM.

To further investigate the potential role of *TXNIP* in regulating RRV infection, we also sought to determine whether variances in *TXNIP* expression were localized to specific immune cell subsets in LN cells. In particular, as RRV naturally infects B cells *in vivo*, serving as a site for RRV replication and latency, it was of interest to assess changes in *TXNIP* expression levels in LN B cells from WT BAC and vCD200 N.S.-infected RM. To achieve this, cells from d0 and d28 pi LN biopsies from each animal were subjected to sequential magnetic bead sorting to obtain purified CD20+, CD14+, and CD3+ cell populations, and RNA was then isolated from these and any remaining unsorted cells. qRT-PCR analysis of *TXNIP* expression levels in individual cell subsets revealed that the largest average decrease in *TXNIP* expression occurred in CD20+ B cells from RM infected with vCD200 N.S. (8.5-fold), which was a higher reduction than that observed in CD20+ B cells from WT BAC-infected RM (3.1-fold) (Fig 7B). This observed difference in B cell *TXNIP* expression levels was found to be statistically significant between infection groups. A slightly larger decrease in *TXNIP* expression in CD3+ T cells was also observed in vCD200 N.S.-infected RM when compared to WT BAC-infected RM, though this difference was not statistically significant, while no difference in reduction of *TXNIP* expression levels was observed in CD14+ cells or unsorted LN cells. Overall, this data demonstrates that in the absence of vCD200, *TXNIP* expression is reduced predominantly in CD20+ B cells in LN tissue of RRV-infected RM. Although the specific mechanism by which vCD200 may help promote *TXNIP* expression is currently unknown, in general, these findings suggest that decreased *TXNIP* expression in vCD200 N.S-infected RM may be associated with the development of higher viral loads in LN tissues of these animals.

## Discussion

To gain a better understanding of the effects of *de novo* KSHV infection and virus-encoded factors on host gene expression patterns, the use of a closely related animal model is critical. RRV infection of RM serves as a well-established nonhuman primate model for the study of KSHV infection, immune regulation, and pathogenesis *in vivo*. Previous studies from our laboratory have investigated the role of the RRV immunoregulatory protein vCD200 on immune responses and viral infection *in vivo*, utilizing a BAC-derived mutant form of RRV lacking expression of vCD200 (vCD200 N.S.). These studies demonstrated that RRV vCD200, a homologue of KSHV vCD200, has negative effects on viral replication and immune responses during *in vivo* infection, suggesting a role for this and other gammaherpesvirus vCD200 molecules in regulation of viral infection and pathogenesis. To date, the only other vCD200 molecule to be examined for functionality *in vivo* is that encoded by myxoma virus M141R, which was found to increase disease severity and inhibit activation of macrophages and T cells in a rabbit infection model of infection [9]. In general, more information is needed to better define how various vCD200 molecules regulate infection and disease development *in vivo*, and specifically, how they may affect host gene expression during infection.

RRV-encoded factors that functionally regulate cellular signaling pathways, such as vCD200, are likely to have effects on cellular gene expression patterns, either directly or indirectly, which could result in a variety of changes in cells affected by RRV infection. As higher replication levels are observed in RM infected with vCD200 N.S. versus WT BAC, we sought to better understand how RRV vCD200 affects cellular gene expression patterns that may ultimately impact viral loads *in vivo*. In addition, a major goal of our studies was to examine global changes in cellular gene expression profiles in LNs from infected RM, to gain a better overall understanding of the effects of *de novo* RRV infection on host gene expression pathways potentially associated with immune modulation, viral replication, and disease development *in vivo*.

Analysis of RNA-seq data indicated that, as expected, RRV gene expression is detectable in d28 pi LN tissues from infected RM, and also revealed that no major variations in viral gene expression patterns existed between WT BAC and vCD200 N.S. infection groups. Although slightly higher levels of viral transcripts were present in vCD200 N.S.-infected LN at d28 pi, this is likely due to the occurrence of higher viral loads in these animals at this timepoint. In both infection groups, genes from the latency-associated region near the right end of the RRV genome displayed the most predominant expression, indicating that the infection is trending towards latency at this timepoint in LNs of RRV-infected RM.

Comparison of changes in cellular gene expression from d0 to d28 pi in LN samples from all eight RM combined, regardless of virus infection group, uncovered a number of host genes altered due to RRV infection. Four of these genes, *CX3CR1*, *RasD1*, *AICDA*, and *GPC1*, were further confirmed by follow up analyses to be differentially expressed after RRV infection, and importantly, have all been suggested to possess oncogenic potential. For example, *CX3CR1*, which encodes the receptor for fractalkine, was found to be upregulated in LN of RRV-infected RM. Fractalkine, the sole member of the CX3C chemokine family, can function as a chemoattractant for monocytes, NK, mast cells, T cells, and B cells expressing the CX3CR1 [55, 56], and although the CX3CL/CX3CR1 signaling axis is involved in normal immune regulation, dysregulation of CX3CR1 signaling has also been found to be associated with variety of human cancers [28]. In particular, the overexpression of CX3CR1 in B cell lymphoma and B-CLL in humans suggests that an upregulation of this receptor due to RRV infection may also be associated with cancer development in RM [26–28]. Alternatively, *RasD1*, which encodes a putative B cell regulatory protein that can inhibit cellular growth and proliferation [29, 30], was identified as being significantly downregulated after RRV infection. Thus, in addition to inducing the expression of genes that may promote abnormal B cell growth and expansion, RRV infection is also capable of downregulating the expression of genes that may repress B cell proliferation. Interestingly, the same animals utilized in this study were previously found to display peak levels of B cell hyperplasia at d28 pi [18], suggesting that alterations in genes that regulate B cell growth may help promote B cell growth after RRV infection.

RRV infection was found to induce *AICDA* expression in LN of RRV-infected RM. Cell sorting and tissue staining analysis also revealed that *AICDA* expression is induced predominantly in B cells of LN tissue at d28 pi, though in some animals elevated expression of *AICDA* was also detectable in other cell types, including T cells. Induction of *AICDA* in LN is part of the development of a normal antibody response, where it promotes somatic hypermutation and class switching in germinal center B cells, and in fact, anti-RRV antibody responses in these animals reached their peak levels near d28 pi [18]. Despite this, elevated or sustained levels of *AICDA* expression in B cells, or aberrant expression in other cell types, also has the potential to promote off target mutations that lead to oncogenesis [33, 34]. Thus, the upregulation of *AICDA* by RRV in LN of infected RM could be a mechanism involved in the development of RRV-related cancers, such as lymphoma. In support of this idea, the induction of AICDA expression has been shown to be associated with EBV infection of B cells, and has been suggested to be directly involved in the development of EBV-associated B cell cancers [57–62]. Further, KSHV infection of B cells *in vitro* can induce the expression of AICDA [63]. Importantly, however, it has not specifically demonstrated that *de novo* infection *in vivo* by either EBV or KSHV is capable of inducing AICDA expression. Our data thus provides further supporting evidence that upregulation of *AICDA* expression may be a common effect associated with infection by oncogenic gammaherpesviruses such as RRV, KSHV, and EBV, which could ultimately promote the development of B cell-derived lymphomas *in vivo*. Further analysis of the role of the specific effects of AICDA induction on RRV-associated disease will ultimately help decipher the relevance of this molecule to cancer development associated with gammaherpesvirus infections. In addition, as EBV proteins LMP1 and EBNA3C have both been shown to be capable of inducing AICDA expression in B cells [57, 64], future studies to define specific RRV genes responsible for induction of AICDA expression during *in vivo* infection will also be of importance.

The gene displaying the highest change in expression overall in our study was *GPC1*, which was found to be induced in LN tissue after RRV infection. Importantly, protein expression analysis of tissue samples from the single animal in the study cohort that developed lymphoma, and which displayed the highest elevation in GPC1 transcript levels in LN biopsy samples, demonstrated that GPC1 protein levels are also elevated in LN B cells during acute RRV infection, and are highly induced in B cells of lymphoma tissue. This finding is particularly intriguing, as GPC1 is a cell surface proteoglycan that is capable of enhancing signaling of cell surface receptors, promoting increased cellular proliferation and has been linked to the development of cancer in various models [37, 38]. Of further significance, GPC1 been found to be enriched in cellular exosomes in several types of cancers [37, 39, 40], and has been explored for potential use as a biomarker for the early detection of cancer development [40]. Thus, upregulation of GPC1 by RRV infection may have the potential to promote increased proliferation and oncogenic potential of RRV-infected cells *in vivo*. In addition, the detection of high levels of this protein in lymphoma tissue strongly suggests that this molecule could in fact be associated with cancer development in RRV-infected RM. Further exploration of the role of GPC1 in RRV-induced cancer development and the possible use of this molecule as a cancer biomarker will provide important insight into the relevance of this molecule to KSHV oncogenesis in humans.

In addition to comparing the effects of RRV infection alone on cellular gene expression profiles, the contributions of RRV vCD200 to gene expression differences in infected RM *in vivo* were investigated. Comparison of WT BAC and vCD200 N.S. infection groups was initiated largely in an attempt to address differences in viral loads observed between WT BAC and vCD200 N.S.-infection groups, in which RM infected with vCD200 N.S. virus displayed higher viral loads early during infection, despite the presence of higher T cell responses against RRV in these animals [18]. As vCD200 is an inhibitory protein that negatively regulates CD200R+ immune cells, it was anticipated that variations in cellular gene expression profiles might exist between WT BAC and vCD200 N.S. infection groups. The identification of any such variations in gene expression could help illuminate mechanisms that regulate viral loads, as well as help elucidate the overall effects of vCD200 *in vivo*. Overall, the number of genes whose expression was altered due to the presence of vCD200 were lower than the number of genes affected specifically due to RRV infection. Of the genes displaying variability in expression between WT BAC and vCD200 N.S. infection groups, only one gene appearing to possess the potential to affect RRV replication, *TXNIP*, was confirmed to be downregulated specifically in LN of vCD200 N.S.-infected RM.

TXNIP is a 46kDa protein that was first identified as a Vitamin D3 up-regulated protein (VDUP1) in human AML cells [65]. TXNIP is a negative regulator of Trx, which directly interacts with and inhibits Trx function [41]. The Trx system is a major cellular reducing system, and is involved in the regulation of maintaining a reducing environment in cells, inhibiting ROS production to prevent cellular damage. Further, TXNIP has been found to affect cellular processes ranging from lipid and glucose metabolism to transcription, promote apoptosis and inflammation, and inhibit cellular proliferation. Indeed, due to inhibitory effects on cell growth, TXNIP is classified as a tumor suppressor [42, 47], and downregulation of TXNIP expression in a variety of human cancers, including B cell lymphoma, has been reported [66–68]. TXNIP expression is known to be regulated by a variety of factors, including cellular stresses, glucose, and cytokines [69–71]. Further, TXNIP expression has been shown to be affected by infection with several oncogenic viruses, including HTLV-I, HBV, HCV, EBV, and KSHV [48–54], and the involvement of TXNIP in viral oncogenesis has also been suggested [52, 53]. In regards to gammaherpesviruses, TXNIP expression has been found to be downregulated by EBV lytic infection in B cells [48, 50], and induced by transgenic expression of LMP2A in mice [51], while in the case of KSHV, latent infection of endothelial cells results in the induction of TXNIP expression [49]. Despite this information, the actual mechanisms of regulation and any potential effects of TXNIP expression on these viruses has not been defined.

Our findings indicate that larger reductions in *TXNIP* gene expression occur in vCD200 N.S.-infected RM, suggesting that vCD200 is capable of promoting *TXNIP* expression during RRV infection. In addition, analysis of sorted LN cells demonstrated that the only significant difference in *TXNIP* expression levels between infection groups occurs in B cells, indicating that vCD200 mainly affects *TXNIP* expression in this cell type. Despite this, the specific mechanism by which RRV vCD200 promotes *TXNIP* gene expression in B cells of RRV-infected LN is unknown, though some possibilities include the activation of signaling pathways in CD200R+ B cells that directly affect *TXNIP* transcription, or alterations in LN tissue cytokine profiles that affect *TXNIP* expression in these cells. As cytokines such as IL-1β and TNFα have been shown to inhibit TXNIP expression [71–73], and our previous studies have demonstrated that RRV vCD200 is capable of inhibiting cytokine production of CD200R+ immune cells [6, 18], it is plausible that elevated levels of cytokines in LN of vCD200 N.S.-infected RM could ultimately result in decreased *TXNIP* gene expression in B cells of these tissues. Despite the mechanism involved in regulation, higher levels of TXNIP expression are likely to have negative overall effects on LN B cells through inhibition of cellular proliferation, promotion of apoptosis, or increased inflammation. Indeed, proliferation of various cell types, including immune cells, have been shown to be inhibited by TXNIP expression *in vitro* [46, 47], and further, germinal centers from *TXNIP* KO mice have been found to possess higher numbers of B cells and increased Ki67 expression levels, suggesting that TXNIP can directly inhibit proliferation of this cell type *in vivo* [74]. As B cells are the primary site of RRV infection and latency *in vivo*, higher levels of TXNIP in this cell type are therefore likely to be broadly inhibitory to RRV replication. In addition, in the case of HCV, TXNIP has been shown to negatively affect virus replication, further indicating the potential significance of this protein to the regulation of viral infections [54]. Overall, this information suggests that the presence of decreased levels of TXNIP expression in LN B cells from vCD200 N.S.-infected RM may at least partly explain the elevated viral loads observed in LN tissue from these animals. Although our preliminary studies have not yet demonstrated a direct effect of TXNIP on RRV replication, future studies aim to determine both the specific mechanism by which vCD200 alters TXNIP gene expression in RRV-infected B cells, and define the effects of TXNIP on RRV replication, both *in vitro* and *in vivo*.

In the context of RRV infection *in vivo*, the precise levels and timing of expression of vCD200 within individual cells of an infected tissue sample is expected to be variable at any given time post-infection. As such, the effects of vCD200-CD200R signaling, and any resulting changes in cytokine profiles or other downstream processes that may ultimately affect global host gene expression within infected animals, will be more subtle than what would be observed in a highly concerted infection analyzed *in vitro*. Indeed, even specific examination of our RNA-seq datasets for genes and gene pathways potentially associated with or affected by vCD200-CD200R signaling did not reveal any genes with statistically significant differences in expression between WT BAC and vCD200 N.S. infection groups. Further, the inherent variability of NHP is a constant factor that affects the response to viral infections, and can impact the ability to define true differences in responses between animals. Taken together, this is likely to explain why fewer differences in gene expression were observed when comparing WT BAC and vCD200 N.S. infection groups (*n* = 4 per group) to pre- and post-infection LN samples from both groups combined (*n* = 8), as more robust changes are likely to be induced within the host simply due to the effects of RRV infection in general, when compared to those resulting from effects of the expression of a single viral immune-regulatory molecule. However, this would also suggest that those genes identified as displaying expression differences in LN tissue samples between WT BAC and vCD200 N.S. infection groups are indeed significant in the context of vCD200 regulation *in vivo*, since the effects of vCD200 on these genes are still detectable in the background of general RRV infection. The analysis of additional timepoints and LN biopsy samples in future studies, combined with RNA-seq analysis of sorted cell populations, will allow for the better definition of the timing and effects of vCD200 expression on specific cell types during RRV infection.

Overall, this study demonstrates the ability to determine changes in viral and cellular gene expression profiles in lymphoid tissues relevant to both RRV- and KSHV-associated disease, such as LN, derived from *de novo* infected RM at defined points pi. This is an invaluable model and approach that can allow for the assessment of changes in both viral and cellular genes after *de novo* infection, and may lead to novel information that will provide a better understanding KSHV pathogenesis in humans. Future gene expression studies will be aimed at examination of more LN biopsy samples and timepoints from RRV-infected RM, as well as tissues such as RF and lymphoma, in order to better define cellular and viral gene expression patterns associated with RRV and KSHV disease development.

## Supporting information

S1 File Viral gene counts from RNA-seq data. (XLSX)

S2 File Differential expression and statistical analysis of normalized cellular gene counts from RNA-seq data. (XLSX)
